# Restoration of Visitors through Nature-Based Tourism: A Systematic Review, Conceptual Framework, and Future Research Directions

**DOI:** 10.3390/ijerph18052299

**Published:** 2021-02-26

**Authors:** Mengyuan Qiu, Ji Sha, Noel Scott

**Affiliations:** 1 College of Economics and Management, Nanjing Forestry University, Nanjing 210037, China; mengyuan881123@gmail.com; 2 College of Business Administration, Jiangsu Vocational Institute of Commerce, Nanjing 211168, China; shaji@griffith-edu.org; 3 Gold Coast Campus, Griffith Business School, Griffith University, Gold Coast, QLD 4222, Australia

**Keywords:** natural environments, nature-based tourism, restoration, systematic review, well-being, sustainable development

## Abstract

Visiting natural environments could restore health and contribute to human sustainability. However, the understanding of potential linkages between restoration of visitors and nature-based tourism remains incomplete, resulting in a lack of orientation for researchers and managers. This study aimed to explore how visitors achieve restoration through nature by analyzing published literature on tourism. Using a systematic review method, this study examined destination types, participant traits, theoretical foundations, and potential restorative outcomes presented in 34 identified articles. A new framework that synthesizes relevant research and conceptualizes the restorative mechanisms of nature-based tourism from a human–nature interaction perspective was developed. Owing to the limitations in the theories, methods, cases, and the COVID-19 pandemic, interdisciplinary methods and multisensory theories are needed in the future to shed further light on the restoration of visitors through nature-based tourism. The findings provide a theoretical perspective on the consideration of nature-based tourism as a public-wellness product worldwide, and the study provides recommendations for future research in a COVID-19 or post-COVID-19 society.

## 1. Introduction

Urbanization, resource exploitation, and lifestyle changes have reduced people’s opportunities for preserving and improving their quality of life [1,2,3]. Furthermore, there exists a wide consensus that the outbreak and severity of the COVID-19 pandemic not only affects physical health but also mental health and well-being. It is most likely that society will face an increase in health challenges, behavioral disturbances, and substance use disorders as extreme stressors exacerbate or induce psychiatric problems [4]. While the pandemic will end eventually due to medical developments, its ill effects on the health and well-being of the general population will remain for a long time [5]. A growing number of people are craving for an opportunity to get close to nature to heal their bodies and minds when facing such a global crisis [6].

The need for restoration through nature has a long history, originating from ancient Chinese healers and Greek philosophers, and the belief that humans can improve their body and mind in natural environments repeatedly appears throughout recorded human history [7]. The process of renewing or recovering physical, psychological, and social capabilities that have become depleted in meeting ordinary adaptational demands is described as the concept of “restoration” [8]. Nature-based tourism, which primarily involves the direct enjoyment of undisturbed natural environments, is an important way for people to recover from stress and mental fatigue [9]. According to the visual characteristics of the restorative environment (e.g., natural color, spatial structural, vegetation coverage, etc.), Bell proposed the “place palette” and believed that spaces with different colors are varied in their restorative effects [10]. Green spaces and blue spaces are the most common natural destinations that may help recover health and well-being [2,10]. Such spaces range from gardens and parks to woodlands and forests as well as oceans, coasts, and inland water bodies, and they also include environments with various natural elements. Travel and tourism make up the largest service industry in the world, while nature-based tourism serves as a primary part of this industry [11]. In 2019, over eight billion people visited nature reserves around the world for relaxation or recovery, generating an estimated revenue of $600 billion [12].

However, while the term “restoration” is occasionally used within tourism theory, it has not been clearly defined, related to nature, and theorized or tested until recently [13,14]. Apart from the number of published empirical studies on the restorative experiences of visitors through nature, no structured overview of research findings on the relationship between visitors and nature-based tourism exists. A general understanding of how visitors achieve restoration through nature-based tourism is not only essential to maintain and improve the well-being of visitors in our rapidly urbanizing world but also provides a more balanced view of the conservation and utilization of nature [15].

With this background, the purpose of this study was to propose a conceptual framework to describe the relationship between restoration of visitors and nature-based tourism through a systematic review. A conceptual framework is defined as a network of linked concepts. It has significant advantages on its capacity for modification and its emphasis on understanding instead of prediction [16]. Before the review, the methodology of creating a comprehensive list of articles pertaining to the associations between restoration of visitors and nature-based tourism is provided. In the review, the results of identifying, selecting, appraising, and synthesizing the evidence for restoration of visitors through nature-based tourism are presented. Subsequently, a conceptual framework of how visitors achieve restoration through nature-based tourism is discussed. To support the conceptual framework, empirical findings relevant to environmental triggers, experience of visitors, and restorative outcomes are analyzed. Finally, directions for future research are discussed.

## 2. Materials and Methods

### 2.1. Systematic Literature Review

This study performed a systematic review of relevant literature that specifically examined the restorative mechanism of visitors in natural environments. Systematic literature reviews (SLRs) differ from other forms of reviews, such as traditional literature reviews and commentaries [17]. A protocol of an SLR is identified a priori and details the study inclusion criteria, establishes a series of review questions, identifies relevant studies, appraises their quality, and summarizes the evidence to provide an overall understanding of the research on a certain topic [18]. The SLR is a suitable method for this study because it synthesizes findings from recent literature while reducing the effect of the reviewers’ own biases, thus identifying research gaps and providing suggestions and directions for further research. Hartig et al. agreed that an SLR is an improved method for the identification of academic evidence and that it is suitable for application in tourism [8]. In this study, we established a three-step system for identifying, selecting, and critically evaluating relevant literature to address the research questions.

### 2.2. Search Strategy and Selection Criteria

The first step of the three-step process involved a comprehensive literature search. We restricted our search to primary research articles in peer-reviewed scientific literature and focused on identifying articles relevant to this review through standardized search methods, including electronic database searches and opportunistic searches through relevant reference lists [19]. Our review considered articles published between 1 January 1989 and 1 October 2021. The year 1989 is important because seminal investigations on restoration were published [20]. The ISI Web of Science (WoS) and Scopus were used as scientific search engines to find appropriate literature. WoS is the most commonly used search engine for literature reviews; however, it does not contain a sufficient number of tourism journals. Scopus was thus used as a complementary database because its coverage of tourism journals is more comprehensive [21]. The search terms were entered using the categories “title, abstract, and keywords” for Scopus and “topic” for WoS. The search profile was based on several primary search terms, which can be divided into two groups: one group referred to the natural environment and the second group to the restorative experience of nature (Table 1). The terms were chosen based on literature review on restoration through nature [8,22,23,24]. In WoS, we used the category of “hospitality, tourism, leisure, and sports” to refine the search for articles. In Scopus, we used the titles of journals to find articles published in the field of tourism.

### 2.3. Study Eligibility Criteria

Second, articles were screened and those articles with titles or abstracts that did not match the main research objectives of our study were excluded. Articles on restoration through nature were included based on the following criteria [22,25]:

1. The article was an original research article or literature review.
2. The article reported data on any measure of restorative experience. The restoration could include psychological health, cognitive rejuvenation, social well-being, or spiritual improvement.
3. The article presented evidence suggesting that visitors are directly exposed to the natural environment. The natural environment was used in a broad sense to include any environment that appeared to be green and blue. “Direct exposure” indicated physical presence within the environment and the use of the environment as a setting for tourism activity. Both observational and experimental studies were included. However, experiences based on virtual environments comprising pictures, slides, or videos were not included as participants were not directly exposed to the real natural environment [25].
4. The article belonged to the field of tourism, hospitality, or leisure, but not sports.

Excluded from the review were studies that focused on the benefits of everyday athletics/exercise performance in natural environments and resilience to natural hazards (e.g., earthquake, debris flow, water, and soil loss) rather than on humans. To reduce assessment bias, the review of relevant literature was shared equally by the two authors. The primary author performed a final check of the selected journal articles to ensure equality in evaluation.

### 2.4. Narrative Analysis of the Selected Articles

Finally, owing to the heterogeneity of the literature selected (in terms of approach, key concepts, designs, and methods), a form of narrative analysis was applied [26]. Narrative analysis, which adopts a textual approach to synthesis, is a widely recognized and validated approach, and it is used when considerable differences in terms of design, methods, outcomes, and analysis exist. Basic information was extracted from all articles that met the review criteria, and the information was input into a standardized spreadsheet, including author names, year of publication, country, study design, study population, sample size, assessment of the environment, types of restorative outcomes measured, confounding factors, and other relevant information, such as information on potential biases. Two authors independently worked on data extraction and evaluation of the quality of the studies. An agreement was reached via consensus and the evidence was classified.

## 3. Results

### 3.1. Overview and General Patterns

**Figure 1:**
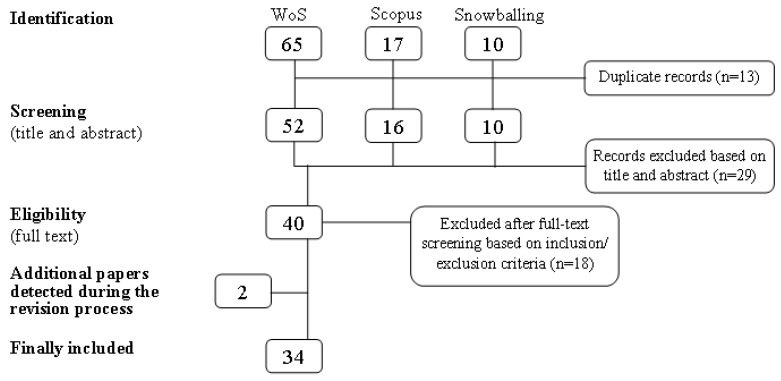
Flow chart of the screening process. WoS, Web of Science.

During the initial search process, 92 studies were identified, 65 using WoS, 17 using Scopus, and 10 using the snowballing system (Figure 1). These studies reflected the widespread discussion on the restorative effects of nature on visitors. Based on screening of the titles and abstracts, 40 journal articles were selected. Most of the articles that were excluded did not meet the inclusion criteria or were not based on primary research. Full-text screening based on the inclusion and exclusion criteria identified a final list of 34 articles that were relevant for this review.

**Figure 2:**
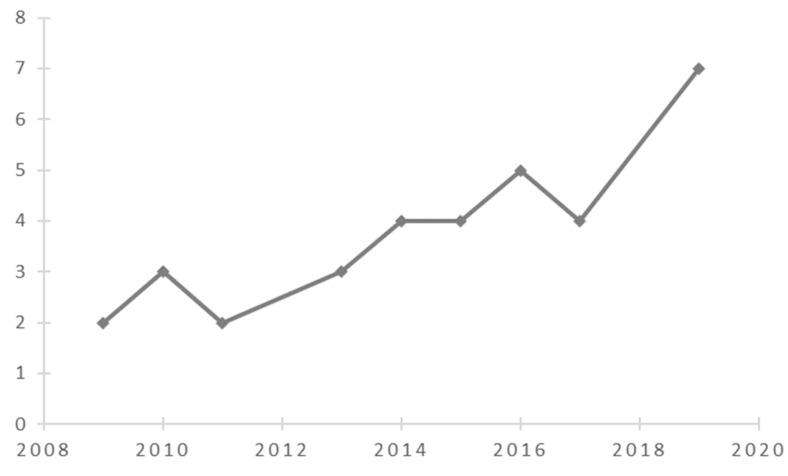
Number of published journal articles meeting inclusion criteria (2008–2020). Note: Year 2020 was included in this figure and in all analyses, even though not all papers from this year are likely to have been published at the time of the review.

**Figure 3:**
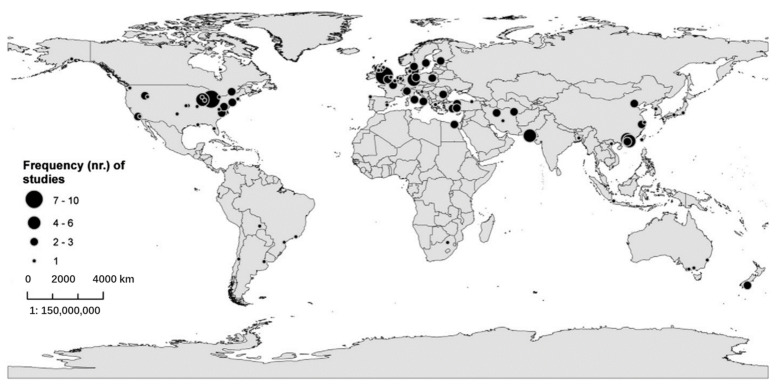
Regional distribution of case studies of this review. Note: Regions are defined by the number of cases identified in them.

Figure 2 shows that since 2009, an increasing number of publications have examined the restorative potential of nature in diverse types of nature-based tourism destinations. This indicates a paradigm shift in terms of restoration research by integrating environment, health, and tourism perspectives [27]. Figure 3 shows that research is dominated by findings from Western developed nations, while Africa and Latin America are poorly represented. The selected articles cover 21 study areas of developed countries or areas, such as the US, UK, Germany, Finland, Japan, South Korea, Hong Kong, and Taiwan. An interesting phenomenon is the increasing number of articles from emerging countries and areas such as mainland China. The progress of research on restoration of visitors through nature-based tourism has been remarkable in these emerging countries in the last decade.

### 3.2. Groups of Publications According to Main Content

According to the narrative analysis, all the articles that met the review criteria were characteristically suitable in terms of design, methods, theoretical background, and outcomes. Based on the focus of the respective articles, they were grouped into the following four “content groups”:

(a) Articles that analyzed the direct and indirect restorative effects of nature-based tourism (articles 1–19).
(b) Articles that considered restoration as an important motivation for nature-based tourism destinations (articles 20–28).
(c) Articles that had a conceptual focus and considered scale development (articles 29–32).
(d) Articles that were literature reviews that specifically focused on restoration in the tourism field (articles 33 and 34).

Table 2 illustrates the structural details of these subgroups with frequencies of studies by country, methods used, and year of publication. Additionally, the theories, target groups, and natural destinations are summarized by screening the basic contents of these studies. Most of the studies focused on the direct and indirect restorative effects of nature-based tourism. Within this group, the number of published studies sharply increased over the last few years. Most of the articles presented studies conducted in Europe and the US, followed by studies from China and Australia. Quantitative statistics with questionnaire-based surveys were the most common method used (58%). Three studies (16%) used a qualitative approach based on semi-structured interviews and coding analysis. Only two studies (11%) adopted physiological experiments to measure the restorative outcomes of visitors participating in nature-based tourism.

The second group of papers dealing with restorative motivations mainly concerned undifferentiated “total” visitors (74%). Only one study directly addressed patients. Most of the studies conducted in China used a questionnaire-based survey as the main method (56%). Studies that combined qualitative and quantitative analyses contributed 22% of the total. The number of published studies decreased gradually with time.

The third group, which discussed methodology development, concerned effective measurement of restorative outcomes. Research in this field has been increasing in the last decade. Most of the studies were conducted in the US and China. Principal component analysis (PCA) and structural equation model (SEM) were the main methods applied in these studies.

The fourth group consisted of literature reviews that specifically focused on restoration of visitors through nature. The papers in this group exhibited disciplinary variations encompassing ecology, epidemiology, psychology, anthropology, public health, and urban/landscape design. Quality analysis was the main method applied in these review articles and some of them used the SLR.

Apart from the two review articles, 32 papers were case studies. The studies mainly focused on visitors to green or blue spaces in urban cities or rural areas, including forests, hot springs, mountains, and coastal areas and beaches (Table 2). Only five papers referred to both local residents and visitors from outside. Three major theories were applied to explain restoration of visitors through nature-based tourism; namely, attention restoration theory (ART) [28,29], stress recovery theory (SRT) [30,31], and the biophilia hypothesis [32]. These theories are multidisciplinary in nature and the details are provided in the next section.

## 4. Discussion

### 4.1. Restorative Triggers: Nature-Based Destinations and Visitors

Restoration is the result of human–nature interaction. As critical triggers, visitors are the subjects in the restorative process, while nature-based destinations provide available resources. A geographical bias toward developed countries in high latitudes was observed in natural destinations, particularly in North America and Europe (Figure 3), probably because only papers in English were included. It is evident from our review that the focus of most of the studies was on visitors from the developed world or restoration through nature in developed countries. However, it is evident from Table 2 and Figure 3 that a significant amount of research also focuses on natural destinations in emerging countries, especially China. As Lehto stated, the specifics of Chinese visitors’ experiences of restoration through nature-based tourism has received growing attention from both academics and practitioners in the last decades [33], as the rapid pace of economic development and globalization has resulted in excessively intense and sub-health conditions. Nature-based tourism has increasingly become a part of the good life for the Chinese for their sustainable development [34].

Most of the studies analyzed refer to undifferentiated “total” visitor groups of nature-based tourism, including local recreationists and outside vacationers. They were often affluent or medium-income earners from developed countries or emerging countries, highlighting a positive relationship between affluence and interest in health and wellness [35,36,37,38,39]. This is unsurprising given the current perceptions of natural areas as a luxury, and it reflects a broader trend identified in travel literature wherein older people and subsistence income earners appear less likely to visit natural environments than younger people and medium-/high-level salary earners [16]. In particular, elderly people from low-income groups struggle to meet the cost of transportation, park entrance fees, and access to recreational facilities in distant natural areas. This trend has been observed in several large cities in France, such as Paris and Marseille [40]. The characteristics of destinations and visitors support the notion that the use of and access to natural areas is socioeconomically driven and varies according to individual circumstances [41].

Nine of the selected 34 journal articles showed that visitors are motivated to visit natural environments, to some degree, by the need to restore their health (Table 3). Getting away from daily routines and life stress is the primary distinguishing motivation for visitors seeking restorative experiences [42]. Health-related motivations are also common among certain types of visitors. Some specific health-related motivations mentioned by various restoration seekers include the improvement of overall health, enhancement of physical attractiveness, rejuvenation of one’s appearance, weight loss, fitness, and curing psoriasis [43,44]. Spa and hot spring destinations were deemed ideal. Apart from physical and psychological restoration, Chan et al. and Dryglas et al. identified enriching one’s travel experience, learning new things about nature, experiencing the beauty of nature, sharing knowledge with others, and enhancing social relationships as spiritual factors that motivate nature-based tourism [39,45].

**Figure 4:**
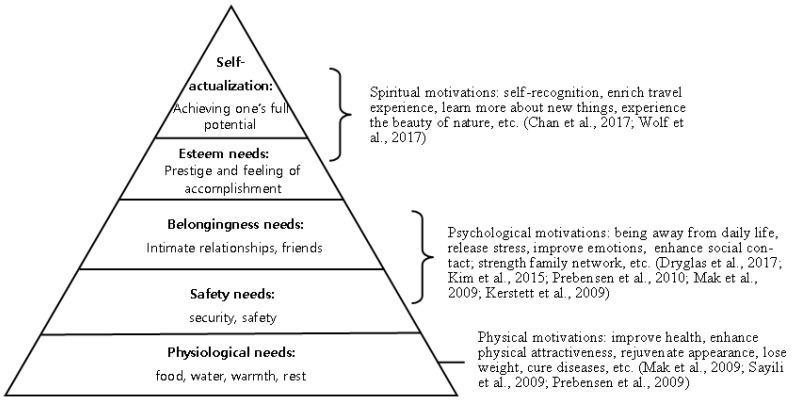
Correspondence between the restorative motivations of visitors in natural environments and Maslow’s hierarchy of needs.

These multidimensional motivations reflect increasing notions within modern societies, suggesting that visiting natural destinations meets diverse needs through physical, psychological, and spiritual improvement [46]. Restoration-related motivations can be explained by Maslow’s hierarchy of needs. Health-related restoration is a physiological need of visitors, which is a basic prerequisite for higher functioning [47]. The primary distinguishing motivation of escape and stress reduction seems to fit most clearly at the psychological level. Strengthening social relationships also fulfills the psychological need for love, affection, and friendship. The highest level of need is that for esteem and self-actualization, wherein an individual fulfills the highest potential and obtains spiritual transformation (Figure 4). Exposure to nature could provide a restoration that fulfills the lower needs and also allows the higher needs of esteem and self-actualization to be met.

### 4.2. Restorative Experience and Its Theoretical Explanation

In tourism literature, ART is the seemingly undisputable explanatory framework for the restorative process. It is a psycho-functionalist theory that distinguishes between directed attention and involuntary attention. After extended use, a visitor’s directed attention may become fatigued and lead to negative emotions and useless behaviors. For functionalists, natural environments seem particularly restorative because they provide an opportunity to “get away” from routine life, contain “fascinating” stimuli that effortlessly engage involuntary attention, allow visitors to be in a large enough world where the “extent” of the environment is perceived, and “compatibility” exists between inclinations of visitors and the environmental demands. These four restorative characteristics—“fascination”, “being away”, “extent”, and “compatibility”—have been used to explain the preference for nature-based tourism and to predict the type of destination that motivates visitors [48]. According to ART, the restorative process is explained well by a general push–pull framework. “Being away” and “compatibility” could be seen as intrinsic factors of visitors to push them to participate in nature-based tourism, while “fascination” and “extent” may serve as extrinsic factors in the destination that pull visitors [14].

SRT is a psycho-evolutionary theory maintaining that because humans have evolved over a long period in natural environments, exposure to certain natural environments automatically elicits a variety of stress-reducing psychophysiological responses [31]. While SRT and ART complement one another, they differ in what drives people toward the restorative nature: in SRT, it is physiological stress, whereas in ART, it is mental fatigue. Attention fatigue can be considered an after effect of stress and may be treated as a condition that increases vulnerability to stress [49]. Biophilia is an evolutionary theory that describes the innately emotional affiliation of human beings to nature. Various empirical studies have suggested that attraction to nature is evidenced across diverse cultures (e.g., [50,51]) and at very young ages (e.g., [52]). The difference between biophilia, ART, and SRT is that the former theory stresses that an environmental preference is an innate part of who we are, while the latter two theories hypothesize that it is affected by people’s need for restoration [53,54,55].

Beyond the idiosyncrasies of each theory, their implications are similar: (1) The properties of nature provide opportunities for visitors to have a restorative experience; (2) Environments perceived as natural tend to be more restorative than those perceived as urban or artificial. In the following sections, we provide a comprehensive review of the empirical research that tested this hypothesis.

### 4.3. Potential Restorative Outcomes and Measurments

**Figure 5:**
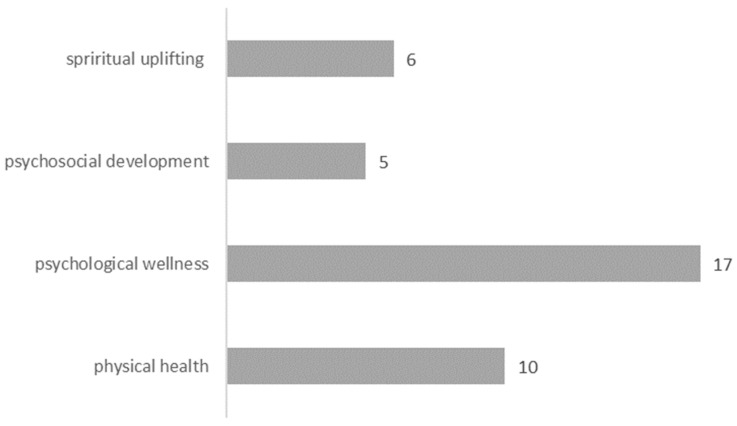
Restorative outcomes from experiences of nature.

The restorative benefits of nature can be divided into physical health, psychological wellness, psychosocial development, and spiritual upliftment (Figure 5). This suggests that restoration through nature-based tourism should not only be limited to recovery from physical fatigue but also refers to a wider range of health benefits to the emotional state, attitude, and behavior [20,27]. The emphasis on mental restoration indicates that with the increase in the discretionary income of visitors, they have shifted their expectations from material products to more personalized socializing experiences [34].

#### 4.3.1. Physical Health and Psychological Wellness

Direct physical health benefits have been reported from physical activity that results from engaging in contact with nature, including healed medical conditions [36,43], contributions to reductions in obesity [39], enhancement of physical fitness [41,56], and general good health [57,58]. Nevertheless, most studies (a total of 17 articles) in our review focused on the potential restorative outcomes of psychological wellness from nature-based tourism. One of the first studies that examined the relationship between mood and nature compared the experience of visitors visiting an urban park and people recreating indoors, and it was published in *Leisure Science* [59]. The study found that the moods of visitors changed slightly but more significantly than that of people indoors, which is consistent with predictions that suggest nature reduces stress. Interestingly, there were few observable differences between sick and healthy visitors with regard to receiving psychological restoration through nature. A substantial body of evidence suggests that visiting natural areas is mentally beneficial to visitors as a whole [34,56]. All 19 articles agreed that natural areas could promote attention recovery and stress relief through high levels of positive emotions, low levels of negative emotions, and a sense of satisfaction with one’s quality of life. For visitors suffering from mood disorders and attention fatigue, immersive natural environments, such as forests with hiking trails, can provide enhanced opportunities for nature connectedness and place attachment and can positively impact the visitors’ mood and feeling of satisfaction [37].

#### 4.3.2. Spiritual Upliftment

Hall pointed out that people may find restoration each day to survive but seek a deep, prolonged restoration when they can afford to take a nature-based vacation [14]. Hence, spiritual upliftment is thought to be the key restorative outcome of nature-based tourism. Spiritual restoration is manifested through values, morals, ethics, and actions of a visitor, and it is at the core of his/her well-being. These positive changes are described as a “transformative process” by Wolf [16], which enables a visitor to increase personal awareness, empathy, and develop new values to become “someone” better than they were. Nature-based tourism embodies relevant properties such as efficacy, power, spirit of place, and existential values that foster transformation, and it can lead to moral development. From witnessing natural wonders, visitors may “become humble before forces greater than them or beyond their control” [60]. Participants of a thematic guided tour in Australian national parks reported a number of behavioral change benefits, including building strong personal relationships, committing to regular exercise, increasing environmental values and stewardship, developing new knowledge and skills, and making physical activity a habit [61]. Pomfret also describes such changes as personal spiritual journeys that visitors experience while participating in adventure activities during their packaged mountaineering holidays [62].

#### 4.3.3. Psychosocial Development

Psychosocial restoration is specifically related to an individual’s development in society and the results of interactions with others [63]. Attention to psychosocial outcomes in tourism literature was given by Shins et al., who studied visitors to forest parks [64]. They classified the psychological outcomes according to the categories of “learning and self/other relations”, “social and self-development”, and “enjoying nature”. Home and Hunziker explored the relationships between each range of 11 activities and a set of 15 possible psychosocial outcomes by developing 11 linear regression models to examine relationships between expected outcomes and frequency of participation in an activity in a green space [65]. The order of psychosocial outcomes that were rated as being most important was similar between this study and that of Shin et al. Dryglas et al. also found that visitors visiting a spa resort in Poland had enhanced opportunities for social contact and could relieve individual isolation [39]. This review suggests that provision of and access to natural environments may ameliorate or even reverse some of these social challenges and ultimately increase social cohesion.

#### 4.3.4. Measures for Restoration through Nature-Based Tourism

Research on restoration through nature often necessitates measurements of recovery. These include explicit measures, such as interviews and questionnaires, and implicit measures, such as psychological monitoring and cognitive tests. Self-report scales are the most common research tool, while most instruments have been developed by researchers that focus on specific restorative properties of a particular environment. Among the nine self-report scales identified in this review (Table 4), Perceived Restorative Scale (PRS) is the most common approach for measuring the restorativeness perceived in nature conducive to visitors [34]. The long history and wide application of PRS demonstrate its generalizability and sensitivity. It has been developed into several versions that have different subscales, items, languages, targeted users, and even item wordings. Perceived Destination Restorative Quality (PDRQ) was used to expand the proposed structure of PRS and survey actual visitors on their restoration through vacation destinations [66]. Although the items of PDRQ used vary from study to study, the four dimensions of PRS (i.e., being away, fascination, extent, and compatibility) are present in each. In the Restoration Scale (RS) used by Han and Huang [67], restoration is demonstrated across emotional, physiological, and cognitive dimensions and generally manifests in behaviors. RS stresses changes in states and capabilities of self-perceived recovery. Restoration Outcome Scale (ROS) includes three dimensions (relaxation and calmness, attention restoration, and cleaning one’s thoughts) or five dimensions (plus subjective vitality and self-confidence). A previous study compared these scales and found that, if restorativeness perceived in nature is conducive to visitors and its mediating effect requires measurement, PRS best fits this approach. However, for measuring the perceived change in psychophysiological and mental restoration, whether as a mediator or an outcome variable, RS is the better choice [67].

### 4.4. The Effects of Restorative Outcomes on Human–Nature Nexus

Multiple benefits arising from nature-based tourism indicate that a visit to natural areas involves a better, healthier, and more sustainable future for both the visitors and the environment. Sloan et al. examined the restorative power of nature in tree house hotels and showed that the physiological and psychological benefits from forest recreation and sleeping in treetops have a positive influence on repeat and future visits [68]. Visitors who took part in nature-based recreational activities more frequently can get more health resources than those who are less involved in nature during their free time. Kim et al. showed that low (e.g., relax and get away from routine) and high order restorations (spiritual benefits) interact with each other to promote the sustainable development of visitors [69]. For example, the positive emotions induced by nature have the potential to strengthen bonds within families and communities through shared park experiences, which in turn builds social capital [70]. The emotional effect of mastering challenges experienced in natural areas yields important benefits for the individual in terms of reducing self-destructive and anti-social behaviors [57] and improving self-esteem and self-confidence, which can also influence spiritual health.

Moreover, Puhakka et al. suggest that the restorative benefits are similar during and after the tourist’s visit to the national park, and it is equal to many popular commercial wellness services, but the range of monetary values is much wider [63]. Accordingly, health and well-being benefits are increasingly used to justify financial and political support for the natural environment and committing to the preservation of biological diversity and ecosystem services [66]. Lehto et al. conducted a survey to understand the functions of restorative outcomes of nature-based tourism in the Chinese context. The research confirms that restoration has positive effects on environmental sustainability by promoting pro-environmental behavior among visitors [33].

### 4.5. Conceptual Framework of Restoration through Nature

**Figure 6:**
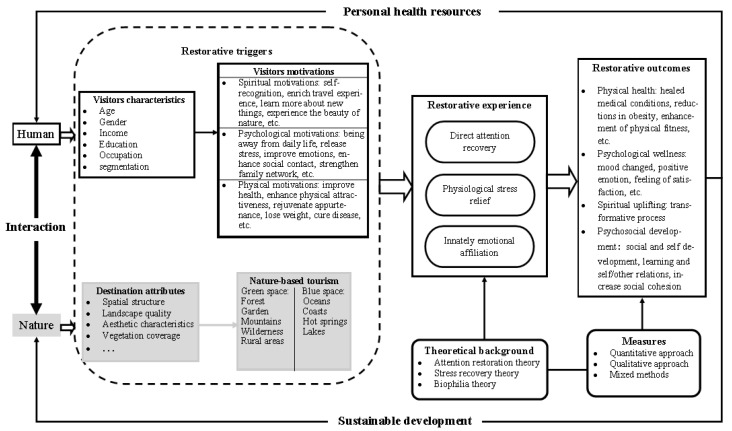
Framework for the restoration of visitors through nature: from a human–nature interaction perspective.

The framework of restoration through nature-based tourism developed in this study shows the human–nature inter-relationships in the context of tourism (Figure 6). Many types of natural destinations provide numerous opportunities for visitors to have contact with nature. In contrast, visitors with different demographic characteristics have variable visitor motivations to push them to visit these natural destinations. When visitors reach natural destinations, the human–nature interaction can induce restorative experiences such as direct attention recovery, physical stress relief, and innate emotional affiliation. These experiences are beneficial for the physical, psychological, spiritual, and psychosocial restoration of the visitors. Therefore, the restorative outcomes arising from nature-based tourism encourage visitors to regard nature as a personal health and well-being resource. Furthermore, these restorative outcomes can raise awareness on committees for the preservation of biological diversity and ecosystem services among the visitors. Thus, nature-based tourism can fulfill the mandate of conserving natural areas while contributing to the area’s sustainability. The conceptual framework echoes the finding of Mannell and Iso-Ahola suggesting that restoration is not passively escaping all perceptions; instead, it activates a more primal and natural mode of perception based on effortless fascination, resulting in recovery and rejuvenation [71].

Most studies to date have only focused on one particular pathway from nature-based tourism to restoration, while few research studies have addressed combinations involving two or more pathways. Our framework synthesizes these studies and conceptualizes the human–nature interaction mechanism to produce restorative outcomes and promote sustainability. In the following sections, we provide a comprehensive review of the empirical research to support this framework.

### 4.6. Challenges in Conceptual Framework

By synthesizing the concepts presented in the selected articles, the conceptual framework was arrived at, and it sheds light on the restoration of visitors through nature; however, challenges remain. First, the human–nature interaction in the conceptual framework is heavily biased toward affluent earners who visit nature in developed countries. This bias may affect the intensities of different types of restorative outcomes because the distribution of biodiversity is spatially structured and cultural and socioeconomic differences between regions may influence responses to interactions with nature [40]. Our understanding of the restoration of visitors with different demographic cohorts in various parts of the world is limited because some specific groups are underrepresented. A comparison of restorative outcomes among specific groups is difficult. Thus, research on restoration through nature is still lacking in depth and we should, therefore, aim to determine where results may be translated from one scale to another, as is done in other multidisciplinary studies [72].

Second, while current theories have made important theoretical contributions, critics have pointed to important limitations in explaining the restorative experience of visitors resulting from nature-based tourism [73]. These limitations stem from weaknesses and perspectives of each theory discussed in Section 4.3, suggesting that a more nuanced approach that builds upon existing theories or develops new theories is required [74]. To summarize:

5. These theories, singularly or in combination, do not conceptualize the full range of restorative experience from nature-based tourism.
6. They do not fully explicate how nature-based tourism, as a unique concept, supports health and well-being.
7. Existing frameworks have largely overlooked the inherently multidimensional, interactive, and multisensorial complexity of the relationship between visitors and nature-based tourism.
8. Additionally, and perhaps most importantly, traditional theories have focused too strongly on the visual form of nature-based tourism by, for example, focusing on what natural destinations look like in terms of color and shape [75].

Therefore, the existing theories lack a clear operationalization process for destination management operators.

Third, in spite of the remarkable growth of using self-report scales to measure restoration, these scales require further examination and testing. The restorative outcomes often lack comparison with physiological and cognitive data from individuals, such as perspiration, skin conductance, muscle tension, blood pressure, heart rate, brain waves, regional cerebral blood flow, and cortisol, adrenaline, epinephrine, and standard concentration tests [56]. Further empirical research is needed to determine whether the recovery reported by visitors is equivalent to their actual restoration. Another challenge posed by the current framework is that most restorative outcomes do not include a control group of individuals with low fatigue for comparison with fatigued individuals. With this paradigm, it becomes impossible to determine whether superior performance after visiting natural areas in fatigued individuals is diagnostic of recovery or whether it signals an entirely different process unrelated to recovery [74].

Finally, the COVID-19 pandemic has caused a calamitous crisis in the tourism industry worldwide, and it has brought nature-based tourism almost to a standstill [76,77]. The spread of stress and depression from the emergence of new infectious diseases has become a growing social problem. In contrast, according to the framework proposed in this study, the restorative outcomes arising from nature-based tourism have positive effects on the development of visitors as well as the natural area’s sustainability. Post-COVID-19, tourism will be different from that before the crisis, and the hope is in tourism developing with a more nature-based focus [78]. Therefore, research on the restorative process of nature-based tourism in the novel context of COVID-19 will play a critical role in promoting both human and environmental sustainability. What are the short- and long-term consequences of the COVID-19 crisis on the conceptual framework of the restoration of visitors through nature? Will visitors restore their health and well-being through nature-based tourism? If so, what does this mean for the sustainable development of the environment?

## 5. Conclusions

A visit to natural areas involves a better, healthier, and sustainable future. Most studies to date have focused only on one pathway for nature-based tourism to restoration of visitors, while few studies have paid attention to a general understanding of the restorative mechanism of nature-based tourism visitors. Partial or superficial phenomena prevent people from knowing the positive effects of nature-based tourism on human-environment sustainability. To fill this gap, this review provides a systematic synthesis and assessment of available literature that examined the potential linkages between restoration of visitors and nature-based tourism. By comparing the research backgrounds, themes, methodologies, and frontiers presented in the identified 34 papers, this study links the theoretical foundations, destination types, participant traits, motivations for restorative experience, and outcomes. A framework that synthesizes relevant research and conceptualizes restoration of visitors through nature is proposed from a human–nature interaction perspective. The findings refute previous arguments that suggest nature-based tourism is infrequent for most people and that it cannot be an effective means of restoration. Our study suggests that nature-based tourism can be regarded as a public-wellness product to improve the health and well-being of visitors. The positive relationships between visitors and nature-based tourism can also raise awareness of visitors on the dependence of human well-being on nature’s well-being [79]. Therefore, they should protect and utilize natural destinations from a sustainability perspective, and thus promote harmony between humans and nature. The challenges presented by the conceptual framework highlight several important future research directions as follows.

First, several general methodological limitations occurred throughout the reviewed literature. Innovative approaches are needed to understand the role and process of nature in promoting human health and well-being [1]. Interdisciplinary research that integrates social, health, and natural sciences is required. A greater emphasis on longitudinal and experimental design, by making use of mixed methodologies that include measurements of established perception surveys and physiological indicators such as electromyography (EMG), electroencephalogram (EEG), blood volume, pulse, and heart rate should be used to obtain transferable and objective results. Moreover, researchers should be wary of translating the findings of studies that have been conducted in specific settings and for defined indicators and subjects into generalized statements.

Second, since visual sense is relatively well understood as a pathway through which the benefits of experiencing nature are delivered, we suggest exploring a rich auditory, haptic, and visual interaction with the natural environment in restoration research. Based on our results, we hope to shed light on the role of sensory inputs in the restorative process. Moreover, the synthesis of multisensory stimuli in a natural environment is crucial because the monotony of stimulation can be a source of stress, and multisensory inputs can drive affordances, which is important for well-being [73]. Rather than focusing on various types of destinations, this approach recognizes that a multisensory interaction with nature underpins the important processes that support restoration of visitors.

Third, future research is needed to deepen the conceptual framework in order to understand the restoration of visitors through nature-based tourism in a COVID-19 or post-COVID-19 society [15]. COVID-19 is the greatest shock to tourism since 1950, but it presents opportunities for the development of nature-based tourism as well [76]. It is assumed that travel behavior will change after the COVID-19 pandemic. To counter this, a new conceptual framework of restoration of visitors through nature-based tourism must be developed. We expect that the new model will facilitate well-being-oriented design parameters for future destinations, which will promote the restoration of the public and foster sustainability of the environment.

## Figures and Tables

**Table 1:** Search terms.

Environmental Terms	Restorative Terms
Natur*; park*; green space; blue space; open space; garden*; horticulture*; wild*; countryside; rural; outdoors; biodiversity; wood*; forest*	restorati*; stress recovery; therap*; well-being; wellness; quality of life; health; attention; fatigue; spirit*

**Table 2:** Characteristics of tourism studies on restoration from nature.

Group	Case study Countries	Methods	Theory Background	Target Group	Types of Nature-Based Tourism
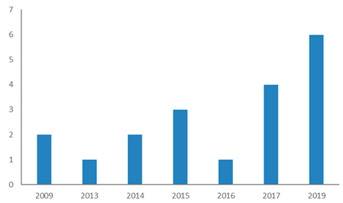 Restorative effects	Europe: 7 US: 4 China: 4 Australia: 2 Japan: 1 South America: 1	Qualitative (interviews, photographs, text analysis, and observation): 3 Quantitative statistics (questionnaire survey, secondary data): 7 Mixed methods: 1 Physiological measures: 2	Attention restoration theory (ART): 12 Stress recovery theory (SRT): 8 Biophilia hypothesis: 3	Outside visitors: 29 Local residents: 5	Forest: 6 Lakes: 3 Mountains: 6 Gardens: 3 Wilderness: 3 Oceans: 2 Beaches: 4 Hot springs: 5
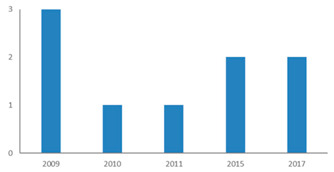 Restorative motivations	China: 4 Australia: 2 Korea: 1 Europe: 1 Turkey: 1	Qualitative (interviews, observation, and focus group): 2 Quantitative statistics (questionnaire survey, secondary data): 5 Mixed methods: 2	Maslow’s hierarchy of needs: 4 Push and pull theory: 2 Optimal arousal theory: 1		
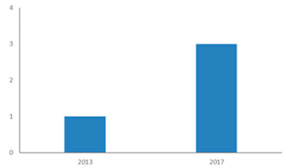 Methodology development	China: 2 US: 1	Quantitative statistics (PCA, SEM): 2	Attention restoration theory (ART): 4 Stress recovery theory (SRT): 3 Biophilia hypothesis: 1		
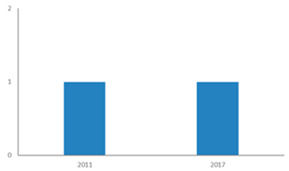 Literature review		Qualitative analysis: 1 Systematic review: 1			

**Table 3:** Motivations of restorative tourism.

Reference	Motivations	Participants	Destinations
Kerstetter et al. (2004)	(1) Improve physical health (2) Observe the land scale (3) Learn new things about nature (4) Experience tranquility	450 ecotourists, 18 years of age and older	Gao-Mei, Guan-Du, Ghi-Gu (wetland and hot spring) in Taiwan, China
Sayili et al. (2007)	(1) Cure psoriasis	104 patients with psoriasis	Kangal Fish Spring (hot spring) in Turkey
Mak et al. (2009)	(1) Relaxation and relief (2) Health and beauty (3) Get away from the pressures of work and social life	302 Spa-goers	Spa destination in Hong Kong, China
Prebensen et al. (2010)	(1) Getting away from pressure and stress (2) Recovering strength (3) Fitness and health	1222 outbound visitors from Norway	Sun and sand destinations in Southern Europe
Moscardo (2011)	(1) Rest and relax (2) Escape normal routine (3) Physical activity (4) Experience the beauty	5540 mass visitors organized by travel operations	The Great Barrier Reef (coastal and sea) in Australia
Yoo et al. (2015)	(1) Close to nature (2) Relaxation (3) Escape form daily routine	21 festival visitors	Goomeri Pumpkin Festival (a festival based on a forest in Australia)
Kim et al. (2015)	(1) Escape from daily life (2) Pursuing a healthy life (3) Enjoying natural environment	430 hiking visitors	Olle Trail located on Jeju Island, South Korea
Chan et al. (2017)	(1) Enrich travel experience (2) Learn more about nature (3) Share knowledge with others (4) Transform lifestyle	430 mainland visitors to Hong Kong, China	Nature-based destinations in Hong Kong
Dryglas et al. (2017)	(1) Improve physical health (2) Release stress (3) Enhance social contact	2050 spa visitors	45 statutory spa resorts in Poland

**Table 4:** Characteristics of the self-report scales.

Source	Scales	Constructs	Items	Language
Pals et al. (2009)	Perceived Restorative Questionnaire Scale (PRCQ)	5-factor structure: fascination; novelty; escape; coherence; compatibility	24	English
Cole and Hall (2010)	Perceived Restorativeness Scale (PRS)	4-factor structure: being away; extent (coherence); fascination; compatibility;	20	English
Hipp and Oguseitan (2011)	PRS	5-factor structure: being away; extent; fascination; compatibility; legibility	26	English
Woran and Arnberger (2012)	PRS	4-factor structure: being away; extent; fascination; compatibility	16	English
Han and Huang (2012)	Restoration Scale (RS)	4-factor structure: emotional; physiological; cognitive; behaviors	8	Chinese
Letho (2013)	Perceived Destination Restorative Quality (PDRQ)	6-factor structure: compatibility; extent; mentally away; physically; discord; fascination	30	English
Korpela et al. (2014)	Restoration Outcome Scale	5-factor structure: relaxation and calmness; attention restoration; cleaning one’s thoughts; subjective vitality; self-confidence	9	English
Letho et al. (2016)	PDRQ	6-factor structure: compatibility; extent; mentally away; physically; discord; fascination	30	Chinese
Chen et al. (2017)	PDRQ	5-factor structure: compatibility; extent; mentally away; physically away; fascination	26	Chinese

## Data Availability

The original data is provided by all the authors. If there are relevant research needs, the data can be obtained by sending an email to Mengyuan Qiu (mengyuan881123@gmail.com). Please indicate the purpose of the research and the statement of data confidentiality in the email.
